# High anti-SARS-CoV-2 antibody seroprevalence in healthcare workers in an Irish university teaching hospital

**DOI:** 10.1007/s11845-021-02690-4

**Published:** 2021-06-30

**Authors:** Ann Leonard, Anna Rose Prior, Phyllis Reilly, Caroline Murray, Meghan O’ Brien, Gillian Maguire, Deborah Ennis, Alex Reid, Ana Rakovac, Gerard Boran

**Affiliations:** 1 Departments of Clinical Chemistry and Laboratory Medicine, Dublin 24 and School of Medicine, Tallaght University Hospital, Trinity College Dublin, Dublin, Ireland; 2 Department of Clinical Microbiology, Tallaght University Hospital, Dublin 24, Ireland; 3 Department of Occupational Health and Wellbeing, Tallaght University Hospital, Dublin 24, Ireland

**Keywords:** Antibody, Healthcare worker, SARS-CoV-2, Seroprevalence

## Abstract

**Introduction:**

Healthcare workers are at very high risk for SARS-CoV-2 exposure and infection. This study evaluated anti-SARS-CoV-2 seroprevalence in healthcare workers in a tertiary care hospital and then correlated seroprevalence with confirmed or suspected SARS-CoV-2 infection in this population since the onset of the COVID-19 pandemic.

**Method:**

The study was approved by our institution’s Joint Research Ethics Committee in June 2020. All volunteers were provided with a consent form, an information leaflet and a questionnaire on the day before phlebotomy. Serum samples were collected from 1176 participants over a 3-month period and analysed using the Elecsys Anti-SARS-CoV-2 assay (Roche Diagnostics GmbH, Mannheim, Germany) which detects total antibodies against the nucleocapsid protein of SARs-COV-2.

**Results:**

Overall anti-SARS-CoV-2 seroprevalence among participating healthcare workers was 17.9%. The rate of confirmed infection by real-time polymerase chain reaction molecular testing prior to participation was 12.2%. Of 211 participants who had a reactive antibody test result, 37% did not have COVID-19 infection confirmed at any point prior to participation in this study, either having had a swab which did not detect SARS-CoV-2 RNA or having never been tested. Seropositivity was the highest (30%) in the youngest quintile of age (20–29 years old). Staff with more patient contact had a higher seroprevalence of 19.5% compared to 13.4% in staff with less patient contact.

**Conclusion:**

This study demonstrates that a substantial proportion of SARS-CoV-2 infections in healthcare workers may be asymptomatic or subclinical and thus potentially represent a significant transmission risk to colleagues and patients.

## Introduction

Healthcare workers (HCWs) are at substantially higher risk of SARS-CoV-2 infection than the general population. This is not only due to exposure to patients with SARS-CoV-2 infection, but also due to a large number of patient-facing professional activities as well as proximity to colleagues and other social interactions in the workplace at a time when the general population was reducing their interactions in line with public health advice [1, 2]. Reports from the Health Protection Surveillance Centre indicate that HCWs represent approximately 6% of confirmed cases of COVID-19 in Ireland to date, and 2% of these were hospitalised [3].

In Ireland, the first case of COVID-19 was identified in February 2020 with cases continuing to rise until a peak of over one thousand cases per day in mid-April 2020. Dublin, the most densely populated county in Ireland, was one of the areas of the country most affected by the pandemic initially. Our hospital, a tertiary referral centre located in Dublin South West with a busy Emergency Department and Intensive Care Unit, has a large catchment area of approximately 640,000 people, serving a large area of Dublin as well as parts of surrounding counties. Dublin South West in particular has one of the higher rates of COVID-19 nationally (https://covid19ireland-geohive.hub.arcgis.com/). Most patients admitted to our hospital live in this locality, as do many of the staff working in the hospital.

The study took place in the Department of Laboratory Medicine; all analyses were completed in the Clinical Chemistry Laboratory with clinical governance of the assay provided by the Microbiology Consultant. The Department of Laboratory Medicine is accredited to ISO 15189; 2012 with standard operating procedures, internal and external quality assurance, state-registered medical scientific staff and appropriate clinical and operational management structure. This study aimed to evaluate SARS-CoV-2 antibody status in healthcare workers (HCWs) in this tertiary care facility and correlate seroprevalence with known or suspected SARS-CoV-2 infection in this population since the onset of the COVID-19 pandemic.

## Methods

### Subjects and samples

This is the first part of a longitudinal seroprevalence study to be conducted over a 12-month period. The study was approved by the Tallaght University Hospital and St James’s Hospital Joint Research Ethics Committee in June 2020. For this first part, samples were collected over a 3-month period from July to October 2020. Participants were sought through the use of the hospital email, all of whom were provided with a consent form, an information leaflet and a questionnaire 24 h prior to their phlebotomy procedure. The questionnaire covered nineteen discrete areas including demographics, vaccination status, vitamin supplementation and possible symptoms of COVID-19 at any point since the onset of the pandemic, previous RT-PCR testing for SARS-CoV-2 infection, and if they had been identified as a COVID-19 contact, either by public health or the occupational health department.

All participants were individually consented by a member of the research team. Participants were required to have been free of symptoms in the 2 weeks prior to sampling.

### Sample collection

Blood samples were drawn into Vacuette® 3.5 ml CAT Serum Sep Clot Activator (Greiner Bio-One) tubes in accordance with the manufacturer’s instructions, standardised phlebotomy procedures and hospital policy. Samples and forms were labelled with a unique study number. Serum samples were allowed to clot for a minimum of 10 min prior to centrifugation; 4000 g for 5.5 min (Hettich ROTIXA 50 RS®) at room temperature. Samples were analysed as soon as possible. In cases of delay in analysis, centrifuged samples were stored between 2 and 8 °C for a maximum of 24 h. If storage was longer than 24 h, samples were stored at ≤ −20 °C. Frozen samples were thawed for a minimum of 1 h at room temperature, vortexed and centrifuged before analysis.

### Analytical procedure

Samples were analysed using the Elecys anti-SARS-CoV-2 assay from Roche Diagnostics, an electrochemiluminescence assay. This test allows the detection of total antibodies (IgG, IgM and IgA) specifically directed against the SARS-CoV-2 nucleocapsid antigen and was performed on the Cobas® e801 module. The test result was reported as reactive or non-reactive based on the recommended cut-off index. According to the manufacturer, a result of <1.0 is deemed non-reactive (negative) and a result of > 1.0 is deemed reactive (positive). The assay has reported sensitivity and specificity of 86.1% and 100%, respectively, after more than 21 days postsymptom onset [4]. All laboratory testing and interpretation were carried out under the stewardship of the Clinical Chemistry Department following which clinical review was completed by the consultant microbiologist (ARP). The assay underwent complete scientific and clinical verification at TUH Clinical Chemistry department. Verification involved precision checks, cut-off index review and internal quality control review and establishment. Further studies involved a four patient population review i.e. two cohorts of patients with known SARS-CoV-2 infection (including serial measurements), specimens bio-banked from June and September 2019 and cohort of inpatients who were not deemed positive for SARS-CoV-2 via RT-PCR. The assay was subject to the internal quality control procedures in place in the Clinical Chemistry laboratory. External Quality Assurance (EQA) was not available at the time of the analysis; however, some samples were sent to external laboratory for proficiency testing. Serology results were compared to RT-PCR testing for SARS-CoV-2 infection. Results were comparable with reported sensitivity and specificity.

Data was entered into Microsoft Excel® 2013 for initial review and results reporting. All data was held in restricted access folders on the hospital network. The following result reporting data was anonymised and transferred to SPSS 26.0 software (SPSS Inc., Chicago, Illinois) for analysis. Seroprevalence was calculated for overall population and defined cohorts A one-way between-group analysis of variance was conducted to explore the impact of age on seroprevalence.

## Results

### Correlation with previous confirmed infection

**Fig. 1:**
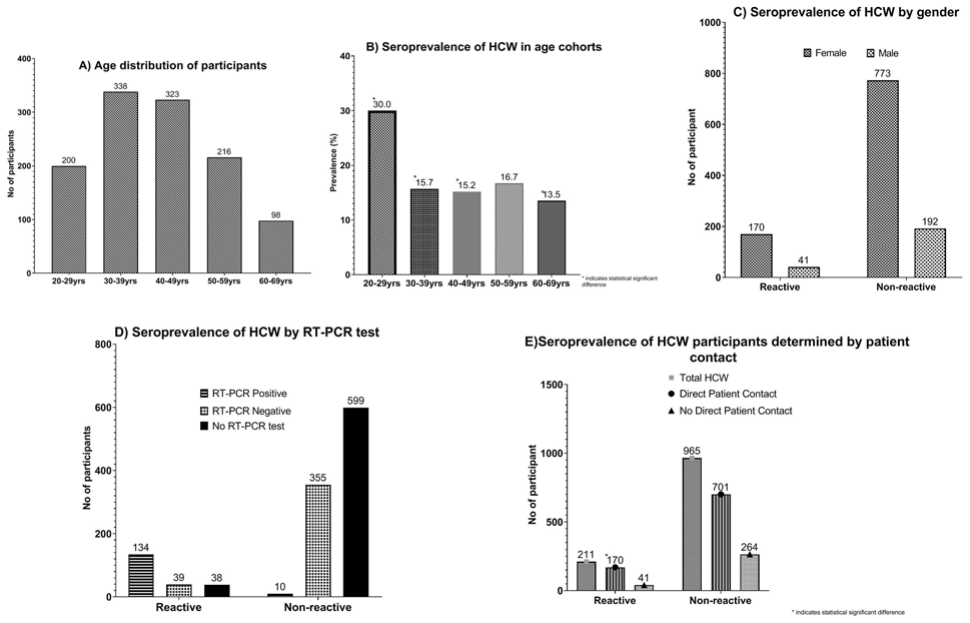
The age distribution of participants and seroprevalence of HCW determined by age, gender, RT-PCR test and patient contact. Individual graphs labelled **A**–**E**. (**A**) Age distribution of participants, (**B**) Seroprevalence of HCW in age cohorts, (**C**) Seroprevalence of HCW by gender, (**D**) Seroprevalence of HCW by RT-PCR test, (**E**) Seroprevalence of HCW participants determined by patient contact

1176 HCWs (39% of staff) were recruited to the study between July and October 2020, consisting of 943 females (80.2%) and 233 males (19.9%). This reflected the gender breakdown of hospital employees which is currently 75% female and 25% male. The age of the cohort ranged from 20 to 69 years old (Fig. 1A). The overall anti-SARS-CoV-2 seroprevalence of the participating HCWs at TUH was 17.9% (n = 211). Seroprevalence was the highest in the youngest cohort with 30% in the age group 20–29 years old (Fig. 1B). This cohort demonstrated statistical significant difference (p < 0.05) from all other age cohorts (30–39 years p = 0.006, 40–49 years, p = 0.006, 60–69 years, p = 0.04) with the exception of the 50–59 year group (p = 0.079). There was no other statistical difference in seroprevalence between age groups. There was no difference between female (18%) and male (17.6%) HCWs (Fig. 1C).

Overall, 45.8% of participants (n = 539) reported having a previous nasopharyngeal swab tested for SARS-CoV-2 RNA by real-time polymerase chain reaction molecular testing (RT-PCR). The rate of confirmed infection by RT-PCR prior to participation in this study was 12.2% (n = 144). Of the ‘RNA not detected,’ participants 41.1% (161) were notified by the public health team of being a direct contact of a confirmed COVID-19 case. Table 1 indicates the symptoms reported by the cohort with positive and negative RT-PCR result. Of the participants who did not have a RT-PCR test, 23.4% (n = 149) reported that they thought they had COVID-19 previously.

**Table 1:** Details of reported symptoms by participants in the study divided into cohorts based on RT-PCR results

Reported symptoms	RT-PCR positive result (N = 144)	RT-PCR negative result (N = 394)	No RT-PCR test (N = 638)	Total
N (%)	144 (12.2)	394 (33.5)	638 (54.3)	1176 (100)
*COVID-19		169	149	318
Diarrhoea	33	34	19	86
Respiratory symptoms e.g. SOB, cough	93	107	87	287
Fever	73	94	64	231
Sore throat	65	118	76	259
Loss of taste/smell	82	24	35	141
Minor respiratory symptoms e.g. runny nose, cold	37	91	56	184
**Attend hospital	23	7	4	34

*SOB* shortness of breath

^*^COVID-19 refers to participants response to in the absence of/negative result to RT-PCR test do you think you had COVID-19?; ^**^Symptoms severe enough required to attend hospital

Table 2 indicates the correlation between antibody detection and previous SARS-CoV-2 RT-PCR results. Of 211 participants who had a reactive antibody test result, 37% did not have SARS-CoV-2 infection confirmed at any point prior to participating in this study, either having had a swab which did not confirm infection or having never been tested (Fig. 1D). 16.6% of those with detectable antibodies reported that they did not think that they had COVID-19 at any point.

**Table 2:** Correlation between SARS-CoV-2 antibody detection and previous RT-PCR test results

Reported results	Reactive SARS-CoV-2 antibody	Non-reactive SARS-CoV-2 antibody	Total
N (%)	211 (17.9)	965 (82.1)	1176 (100)
Nasopharyngeal swab: SARS-CoV-2 RT-PCR detected	134	10	144
Nasopharyngeal swab: SARS-CoV-2 RT-PCR not detected	39	355	394
Not previously tested by RT-PCR	38	600	638

Of those 394 participants with a previous PCR nasopharyngeal swab who did not have SARS-CoV-2 RNA detected, nearly half (n = 169, 42.8%) reported that they thought they had previous COVID-19, either due to symptoms (n = 143) or due to close contact with a confirmed case (n = 26, see Table 1). Antibodies to SARS-CoV-2 were detectable in 39 (9.9%) of them. Of the cohort never tested by nasopharyngeal RT-PCR test, 6% (n = 38) had a positive antibody test (Table 2; Fig. 1D). Of those, 50% reported suspected previous COVID-19 either due to symptoms or close contact with a confirmed case.

### Patient contact

The participants were further divided into groups of HCWs that had greater levels of direct patient contact compared to those with less direct patient contact. Three-quarters (74.1%) of participants worked in roles with direct patient contact (e.g. medical, nursing, social work, catering), and 25.9% were in roles involving less patient contact (e.g. laboratory, administration, management). There was a significant difference in seroprevalence between these groups: 19.5% in staff with more patient contact as compared to 13.4% in staff with less contact (p = 0.022, see Fig. 1E).

## Discussion

This first part of our longitudinal seroprevalence study demonstrated the following key findings:

- HCWs are at a significant risk of SARS-CoV-2 acquisition
- SARS-CoV-2 seroprevalence among a representative cohort of HCWs in our facility was substantially higher than that of the general population following the first wave of the SARS-CoV-2 pandemic in Ireland.
- Approximately, a third of those with detectable antibodies did not have confirmed SARS-CoV-2 infection prior to participation, highlighting the need for ongoing caution and strict adherence to infection control and occupational health guidance to limit spread of asymptomatic infection, particularly at times of high community prevalence.

A national seroprevalence study conducted between June and July 2020 found that 3.1% of participants in Dublin had antibodies to SARS-CoV-2, and the overall estimated national prevalence rate was 1.7% [5]. Overall, the rate of seropositivity of 17.9% detected in our study is much higher than reported in the general Irish population, confirming the higher exposure risk of HCWs. This is similar to that reported by the PRECISE study at St James’ Hospital (SJH), Dublin (15%) [6]. Even for HCWs with less patient contact, seroprevalence was higher than that of the community, possibly reflecting a large number of professional and social interactions in the workplace. This is further supported by another study which demonstrated the rate of seropositivity in HCWs to be 22 times that of the general population [7]. HCWs with direct patient contact demonstrated a significantly higher rate of seropositivity at 19.5%. These findings were in line with those reported by the PRECISE study, which reported a seroprevalence of 21% among nursing staff at St. James’ Hospital, Dublin [6]. The PRECISE study reported that the main risk factor for HCW infection is community prevalence.

Although it is assumed that HCWs are at a higher risk of infection, other studies have reported varying HCW seropositivity compared to the community [8–11]. Our seroprevalence is also significantly higher than reported by similar healthcare institutions in areas that also implemented widespread community restrictions [8, 9]. At various times, particularly early in the onset of the pandemic, there were concerns regarding supply and availability of personal protective equipment. At no point was supply to our hospital interrupted, and PPE was available at all times. However, recommendations around the use of PPE changed at various points. At the start of the pandemic, before the role of transmission from asymptomatic/presymptomatic cases was fully recognised, HCWs donned PPE only when caring for suspected/confirmed cases of COVID-19 in line with infection prevention and control guidelines at that time [12]. Both HCWs and patients inadvertently became infected from asymptomatic individuals. Based on our own local experience and in line with clear evidence of presymptomatic transmission reported in the literature [13], the use of PPE for all patient interactions was soon mandated locally on 23rd March 2020. This was later also recommended in national guidance [14]. Our study did not aim to identify whether the HCW acquired infection in the workplace or the community; however, this distinction is less relevant when it comes to highlighting the risk of infection transmission from infected HCWs to patients or colleagues.

A significant proportion of those HCWs with detectable antibodies did not recall symptoms consistent with previous infection. These could be due to recall difficulties at the time of the study, or due to under recognition of minimal symptoms.

As demonstrated in our study, a substantial proportion of SARS-CoV-2 infections in HCWs may be asymptomatic or subclinical. This potentially represents a significant transmission risk to their colleagues and patients [13, 15]. Approximately, a third of those with positive antibodies did not have confirmed COVID-19 prior to participation, with a half of these not having previously suspected infection. During a period of increased workload and longer working hours, it is possible that mild clinical symptoms may go unnoticed or be mistaken for work-related fatigue. Early recognition of symptoms, particularly low-grade symptoms, with exclusion from work, physical distancing, hand hygiene and appropriate use of PPE at all times remains crucial throughout the course of this pandemic to control ongoing spread of the SARS-CoV-2. Another consideration is the changing case definitions for possible COVID-19. At our facility, there has always been good availability of access to testing for staff. However, due to changing case definitions, earlier in the pandemic, staff may not have been tested as they did not meet relevant criteria at that time. In particular, loss of taste and smell was not widely recognised as a symptom of infection until late March 2020. A number of our staff reported having experienced this early on without undergoing PCR testing.

Molecular tests, such as RT-PCR to detect SARS-CoV-2 RNA, are commonly employed in laboratories as the gold standard to detect cases of COVID-19, and this timely molecular testing is crucial as part of a multimodal strategy to control spread of infection. However, test sensitivity can vary depending on quality of specimen obtained, and timing of specimen collection in relation to onset of symptoms and testing platform and assay employed. Test sensitivity for SARS-CoV-2 molecular assays performed on upper respiratory samples is reported to be in the range of 60–70% [16]. Given the limitations of SARS-CoV-2 RT-PCR testing, it is important that the pretest probability of infection is considered even the most sensitive assay from a good quality specimen will not detect all infections. Furthermore, occupational health advice must be adhered to if staff has symptoms of an acute respiratory illness, even where SARS-CoV-2 has not been detected on a nasopharyngeal swab. Consideration should be given to follow-up antibody testing where available as part of a clinical assessment. Given the absence of other respiratory viruses in widespread circulation, there was a significant likelihood that any reported respiratory symptoms were due to SARS-CoV-2.

This study confirms the importance of consistent use of standard and transmission-based precautions when caring for all patients during this pandemic. These results demonstrate that a high proportion of SARS-CoV-2 infection in HCWs goes undetected, either as it is not detected on PCR testing of nasopharyngeal swabs or staff has had subclinical or asymptomatic infection at some point throughout the pandemic. This supports the requirement as per Irish guidelines [14] for universal mask use for all patient interactions during the pandemic, both to protect staff but also to minimise the risk of staff unknowingly transmitting infection to patients [2].

Our study has a number of limitations. Firstly, staff with previous infection or symptoms may have been more likely to enrol in study thus resulting in a bias. Secondly, as this study took place a number of months following the onset of the pandemic, staff recall of previous low-grade symptoms may have been affected. Thirdly, this study did not aim to distinguish whether infection exposure took place in the workplace (with transmission occurring from either patients or colleagues) or in a community setting. Lastly, it included a large cohort of participants with varying exposure risks in the healthcare setting which were not evaluated.

In conclusion, we demonstrated a substantially higher SARS-CoV-2 seroprevalence among a large group of HCWs in our facility compared to national seroprevalence. HCW are at greater risk of infection due to clinical exposure, particularly where infection is not suspected. In addition, during a period of community restrictions, HCWs have an increased number of workplace interactions compared to the general population.

## Declarations

### Guarantor

ARP and GB.
